# Prevalence of mental illness among COVID-19 survivors in South Korea: nationwide cohort

**DOI:** 10.1192/bjo.2021.1001

**Published:** 2021-10-01

**Authors:** Hye Yoon Park, In-Ae Song, So Hee Lee, Min young Sim, Hong Sang Oh, Kyoung-Ho Song, Eun-Seung Yu, Hye Youn Park, Tak Kyu Oh

**Affiliations:** Department of Psychiatry, Seoul National University Hospital, South Korea; Department of Anesthesiology and Pain Medicine, Seoul National University Bundang Hospital, Republic of Korea; Department of Psychiatry, National Medical Center, Republic of Korea; National Center for Disaster Trauma, National Center for Mental Health, Republic of Korea; Division of Infectious Disease, Department of Internal Medicine, Armed Forces Capital Hospital, Republic of Korea; Division of Infectious Disease, Department of Internal Medicine, Seoul National University Bundang Hospital, Seoul National University College of Medicine, Republic of Korea; Department of Counseling Psychology, the Cyber University of Korea, Republic of Korea; Department of Psychiatry, Seoul National University Bundang Hospital, Republic of Korea; Department of Anesthesiology and Pain Medicine, Seoul National University Bundang Hospital, Republic of Korea

**Keywords:** Anxiety disorders, depressive disorders, epidemiology, post-traumatic stress disorder, risk assessment

## Abstract

**Background:**

Mental illness among survivors of coronavirus disease 2019 (COVID-2019) during the post-illness period is an emerging and important health issue.

**Aims:**

We aimed to investigate the prevalence of mental illness and the associated factors for its development among COVID-2019 survivors.

**Method:**

From 1 January to 4 June 2020, data were extracted from the National Health Insurance Service COVID-19 database in South Korea. Patients with COVID-19 were defined as those whose test results indicated that they had contracted the infection, regardless of disease severity. COVID-19 survivors were defined as those who recovered from the infection. The primary end-point was the development of mental illness, which was evaluated between 1 January and 1 December 2020.

**Results:**

A total 260 883 individuals were included in this study, and 2.36% (6148) were COVID-19 survivors. The COVID-19 survivors showed higher prevalence of mental illness than the control group (12.0% in the COVID-19 survivors *v.* 7.7% in the control group; odds ratio (OR) = 2.40, 95% CI 2.21–2.61, *P* < 0.001). Additionally, compared with the control group, the no specific treatment for COVID-19 group (OR = 2.23, 95% CI 2.03–2.45, *P* < 0.001) and specific treatment for COVID-19 group (OR = 3.27, 95% CI 2.77–3.87, *P* < 0.001) showed higher prevalence of mental illness among survivors.

**Conclusions:**

In South Korea, COVID-19 survivors had a higher risk of developing mental illness compared with the rest of the populations. Moreover, this trend was more evident in COVID-19 survivors who experienced specific treatment in the hospital.

## Background

On 11 March 2020, the World Health Organization declared the coronavirus disease 2019 (COVID-19) crisis to be a pandemic.^1^ The worldwide administration of the vaccine began on 8 December 2020.^2,3^ However, considering the production volume and the speed with which vaccines can be administered, achieving herd immunity may take some time.^4^ Thus, COVID-19 is still being considered an important global health crisis.

Approximately 2.2% of individuals worldwide who contracted COVID-19 died because of the infection,^5^ meaning that 97.8% of people who contracted COVID-19 are survivors. This shows that quality of life of the survivors and sequelae have emerged as important public health issues.^6^ Psychological sequelae has been reported as an important issue in current literature.^7–9^ In a previous cohort study from the USA, survivors of the diseases who had no previous psychiatric history had a higher risk of developing psychiatric disorders during the 14–90 days follow-up period.^10^ In South Korea, a high association of psychological sequelae in COVID-19 survivors has been reported in previous studies.^8,9^ However, to date, studies have only evaluated the total prevalence of mental illness during a short-term follow-up period of 1 month.^9,11^ Thus, there is an urgent need to evaluate the overall mental illness levels with a longer follow-up period.

## Aims

This study investigates the prevalence of mental illness and the associated factors for its development in COVID-19 survivors. It hypothesises that COVID-19 survivors are more likely to develop mental illness compared with other individuals. Moreover, we hypothesised that COVID-19 survivors who experienced specific treatment for the infection in a hospital might have a higher prevalence of mental illness compared with other survivors.

## Method

As a population-level cohort study, guidelines from the Strengthening the Reporting of Observational Studies in Epidemiology have been followed.^12^ The protocol of this study was approved by the Institutional Review Board of the Seoul National University Bundang Hospital; approval number: X-2009-636-902. The National Health Insurance Service (NHIS) sharing service also approved the study protocol and permitted the use of the NHIS COVID-19 database (NHIS-2021-1-070). The need for informed consent was waived because the data in this study were analysed retrospectively in an anonymised form from the NHIS database. The authors assert that all procedures contributing to this work comply with the ethical standards of the relevant national and institutional committees on human experimentation and with the Helsinki Declaration of 1975, as revised in 2008.

### NHIS COVID-19 database

The NHIS COVID-19 database has been created for purposes of medical research in cooperation with the Korea Disease Control and Prevention Agency (KDCA) and the NHIS. The KDCA provided data for all individuals whose polymerase chain reaction (PCR) test indicated a positive result, confirming that the individual had contracted the infection for the period of 1 January to 4 June 2020. These data included date of confirmation and results of treatment in addition to demographic information. The NHIS extracted the control population through stratification methods based on age, gender and location of their home, using ZIP code as of February 2020. Next, the KDCA provided the data on individuals who received a PCR test, and the result was negative. Therefore, the NHIS COVID-19 database contains data for three groups: a COVID-19-positive group; a control population; and PCR negative-tested individuals.

As the only public health insurance system, the NHIS possesses information regarding all disease diagnoses using ICD-10^13^ codes and any drug prescription and/or procedure information. The NHIS COVID-19 database was first developed on 26 June 2020, and last updated on 1 December 2020. The NHIS COVID-19 database had no missing data except for the annual income level for some individuals.

### Study population

The COVID-19 survivors in this study are defined as individuals who were diagnosed with COVID-19 and then discharged from hospital after treatment. Additionally, individuals who were diagnosed with COVID-19 but were not admitted to hospital because they only had mild or no symptoms are also considered as COVID-19 survivors, if they recovered from the infection. In South Korea, individuals who were diagnosed with COVID-19 were admitted to hospital if they had severe symptoms, such as pneumonia. However, if they had no symptoms or only had mild symptoms, they were isolated with close monitoring in specific government-managed centres.

The other population in the NHIS COVID-19 database has been defined as the control group in this study, which consisted of the control group and PCR test-negative individuals. Among the study population, individuals under the age of 20 were excluded because the prevalence and risk factors for mental illness are different in children and adolescents.^14^ In addition, individuals who had a previous history of mental illness up until 31 December 2019, were excluded from the analysis to focus on newly developed mental disorders in 2020.

### Endpoint: development of mental illness

The primary end-point of this study was the development of mental illness. To define mental illness using ICD-10 codes, the criteria used by Lee et al^15^ were used. Mental illness includes non-affective psychotic disorders (F20–24 and F28–29), affective psychotic disorders (F25, F30–31, F32.3 and F33.3), anxiety and stress-related disorders (F40–48), alcohol or drug misuse (F10–16 and F18–19), mood disorders without psychotic symptoms (F32–34, F38–39, excluding F32.3, F33.3), eating disorders (F50) and personality disorders (F60–63 and F68–69). The development of mental illness among the study population was determined according to registered ICD-10 codes from 1 January to 1 December 2020. Thus, the diagnosis of mental illness among the study population was determined in the approximately 6-month follow-up time as the data were from 1 January to 4 June 2020. In South Korea, the ICD-10 codes of mental illness must be registered by physicians or psychiatrists in order for patients to receive financial support for the treatment of mental illness.

### Confounders

The following information was collected and considered as confounders: demographic variables (gender and age), residence and annual income level in 2020, underlying disability (registered on the NHIS database) and Charlson Comorbidity Index (CCI) score, which was calculated using registered ICD-10 diagnostic codes during the 4 years from 2015 to 2019 (Supplementary Table 1 available at https://doi.org/10.1192/bjo.2021.1001). All individuals were divided into seven groups according to age (20–29, 30–39, 40–49, 50–59, 60–69, 70–79 and ≥80 years), and residence was divided into five areas (Seoul, Gyeonggido, Daegu, Gyeongsangbukdo and other areas). In South Korea, the annual income level of all individuals must be registered in the NHIS database to determine individual insurance premiums, which is divided into quartiles (Q1, Q2, Q3, Q4 and unknown).

Additionally, treatment information for all patients with COVID-19 was collected to reflect severe COVID-19 infection similar to a previous study,^16^ including supplemental oxygen therapy, mechanical ventilator use, continuous renal replacement therapy, extracorporeal membrane oxygenation use and high-flow oxygen therapy. Thus, if the survivors were given any specific treatment based on the treatment information, they were considered to be a part of a specific treatment group.

### Statistical methodology

The clinicoepidemiological characteristics of all individuals in the database are presented as mean values with s.d.s for continuous variables (CCI) and as numbers with percentages for categorical variables (all other variables except for CCI). To compare the characteristics between COVID-19 survivors and the control group, a *t*-test and chi-square test were used for continuous and categorical variables, respectively. Subsequently, uni- and multivariable logistic regression analyses were performed to examine if COVID-19 survivors were associated with a higher prevalence of development of mental illness in 2020, compared with the control group.

In the multivariable model (model 1 as main analysis), all covariates were included in the adjustment. Additionally, the COVID-19 survivors were divided into two groups (no specific treatment for COVID-19 group and specific treatment for COVID-19) and included in model 2 to examine the impact of treatment experience on the development of mental illness. Second, the duration of isolation because of COVID-19 was included in model 3 as a continuous variable. A multivariable logistic regression model was used to analyse the development of mental illness; this excluded the individuals who died in 2020, as the deaths during this period (evaluation period) could affect the development of mental illness in both the COVID-19 survivor group and the control group.

Next, for the competing risk analyses, mental illness was divided into seven types: non-affective psychotic disorders, affective psychotic disorders, anxiety and stress-related disorders, alcohol or drug misuse, mood disorders without psychotic symptoms, eating disorders and personality disorders. Seven multivariable logistic regression models were constructed using all covariates to examine which form of mental illness was mostly observed in COVID-19 survivors compared with the control group. Finally, subgroup analyses were performed using multivariable models according to gender (male and female), age (20–39, 40–59 and ≥60 years old), and CCI (0–2 and 0–3 groups). All multivariable models confirmed that there was no multicollinearity between the variables with a variance inflation factor of <2.0, and Hosmer–Lemeshow statistics were used to confirm that the goodness of fit of the models was appropriate at *P* > 0.05. The results of the logistic regression analyses are provided as odds ratios (ORs) with 95% CIs. R software (version 4.0.3; R Foundation for Statistical Computing, Vienna, Austria) was used for all statistical analyses, and a *P*-value of <0.05 was determined as statistically significant.

## Results

### Study population

**Fig. 1:**
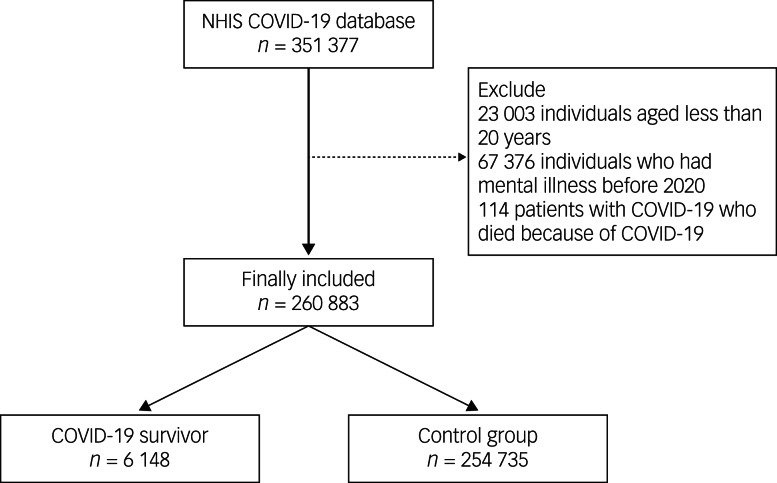
Flow chart for the study. COVID-19, coronavirus disease 2019; NHIS, National Health Insurance Service.

A flow chart depicting the individual selection process in this study is presented in Fig. 1. The NHIS COVID-19 cohort initially included 351 377 individuals. We then excluded from the analysis 23 003 individuals aged less than 20 years, 67 376 individuals who had a mental illness before 2020, and 114 patients with COVID-19 who died from the infection during their hospital admission. Therefore, 260 883 individuals were included in the analysis. Among these, the number of COVID-19 survivors was 6148 (2.36%), and there were 254 735 individuals in the control group. The clinicoepidemiological characteristics of the participants are presented in Supplementary Table 2.

Among the total study population, 20 262 (7.8%) participants were diagnosed with mental illness, and anxiety and stress-related disorders were the most common and were observed in 13 736 (5.3%) participants, followed by mood disorders without psychotic symptoms in 8089 (3.1%) participants. The results of the comparison of clinicoepidemiological characteristics between COVID-19 survivors and the control group are presented in Table 1.

**Table 1:** Comparison of clinicoepidemiological characteristics between COVID-19 survivor and the control group

Variable	COVID-19 survivor *n* = 6148	Control *n* = 254 735	*P*
Gender male, *n* (%)	2414 (39.2)	137 977 (54.2)	<0.001
Age, *n* (%)			
20–29	1916 (31.2)	62 109 (24.4)	<0.001
30–39	748 (12.2)	44 794 (17.6)	
40–49	894 (14.5)	40 263 (15.8)	
50–59	1229 (20.0)	42 337 (16.6)	
60–69	827 (13.5)	31 875 (12.5)	
70–79	369 (6.0)	19 093 (7.5)	
≥80	165 (2.7)	14 264 (5.6)	
Residence, *n* (%)			
Seoul	458 (7.4)	42 646 (16.7)	<0.001
Gyeonggido	369 (6.0)	45 719 (17.9)	
Daegu	4001 (65.1)	79 501 (31.2)	
Gyeongsangbukdo	621 (10.1)	20 326 (8.0)	
Other area	699 (11.4)	66 543 (26.1)	
Residence, *n* (%)			
Q1 (lowest)	1737 (28.3)	54 415 (21.4)	<0.001
Q2	1224 (19.9)	51 866 (20.4)	
Q3	1317 (21.4)	64 048 (25.1)	
Q4	1770 (28.8)	79 920 (31.4)	
Unknown	100 (1.6)	4486 (1.8)	
Underlying disability (registered on the NHIS database), *n* (%)			
Mild to moderate	177 (2.9)	10 019 (3.9)	<0.001
Severe	96 (1.6)	6432 (2.5)	<0.001
Charlson Comorbidity Index, mean (s.d.)	2.7 (2.8)	2.1 (2.2)	<0.001
Comorbid conditions, *n* (%)			
Myocardial infarction	119 (1.9)	6505 (2.6)	0.002
Congestive heart failure	259 (4.2)	19 328 (7.6)	<0.001
Peripheral vascular disease	755 (12.3)	38 200 (15.0)	<0.001
Cerebrovascular disease	460 (7.5)	256 486 (10.0)	<0.001
Dementia	211 (3.4)	10 652 (4.2)	0.004
Chronic pulmonary disease	2982 (48.5)	138 058 (54.2)	<0.001
Rheumatic disease	485 (7.9)	22 605 (8.9)	0.007
Peptic ulcer disease	2318 (37.7)	107 902 (42.4)	<0.001
Mild liver disease	2292 (37.3)	104 838 (41.16)	<0.001
Diabetes without chronic complication	1164 (18.9)	59 722 (23.4)	<0.001
Diabetes with chronic complication	329 (5.4)	18 860 (7.4)	<0.001
Hemiplegia or paraplegia	61 (1.0)	3313 (1.3)	0.034
Renal disease	98 (1.6)	9444 (3.7)	<0.001
Any malignancy	402 (6.5)	31 818 (12.5)	<0.001
Moderate or severe liver disease	18 (0.3)	1717 (0.7)	<0.001
Metastatic solid tumour	48 (0.8)	6045 (2.4)	<0.001
AIDS/HIV	7 (0.1)	421 (0.2)	0.325
Mental illness development in 2020, *n* (%)	738 (12.0)	19 524 (7.7)	<0.001
Non-affective psychotic disorders	13 (0.2)	755 (0.30)	0.224
Affective psychotic disorders	69 (1.1)	2845 (1.1)	0.968
Anxiety and stress-related disorder	489 (8.0)	13 247 (5.2)	<0.001
Alcohol or drug misuse	14 (0.2)	665 (0.3)	0.612
Mood disorders without psychotic symptoms	343 (5.6)	7746 (3.0)	<0.001
Eating disorders	2 (0.0)	167 (0.1)	0.315
Personality disorders	2 (0.0)	57 (0.0)	0.601

COVID-19, coronavirus disease 2019.

The proportion of men in the COVID-19 survivors was higher (39.2%; 2414/6148) than that in the control group (54.2%; 137 977/254 735) with a *P* < 0.001. The proportion of ≥80-year-olds and 70- to 79-year-olds in the COVID-19 survivors were lower (2.7% (165/6148) and 6.0% (369/6148), respectively), compared with the control group (5.6% (14 264 of 254 735) and 7.5% (19 093 of 254 735), respectively). The prevalence of mental illness in 2020 was higher in the COVID-19 group (738/6148, 12.0%) than in the control group (19 524/254 735, 7.7%, *P* < 0.001).

### Development of mental illness in 2020

The results of the logistic regression analysis for the development of mental illness in 2020 are presented in Table 2. In model 1, the prevalence of mental illness in the COVID-19 survivors was 2.40-fold higher than that in the control group (OR = 2.40, 95% CI 2.21–2.61, *P* < 0.001).

**Table 2:** Logistic regression analysis for development of mental illness in 2020^a^

Variable	Development of mental illness, *n* (%)	Logistic regression, odds ratio (95% CI)	*P*
Unadjusted (univariable analysis)			
Control	19 524/254 735 (7.7)	1	
COVID-19 survivors	738/6 148 (12.0)	1.64 (1.52–1.78)	<0.001
Covariates-adjusted model 1 (multivariable analysis)			
Control	19 524/254 735 (7.7)	1	
COVID-19 survivors	738/6148 (12.0)	2.40 (2.21–2.61)	<0.001
Covariates-adjusted model 2 (multivariable analysis)			
Control (*n* = 254 735)	19 524/254 735 (7.7)	1	
No specific treatment for COVID-19 (*n* = 5256)	526/5256 (10.0)	2.23 (2.03–2.45)	<0.001
Specific treatment for COVID-19 (*n* = 892)	212/892 (23.8)	3.27 (2.77–3.87)	<0.001
Covariates-adjusted model 3 (multivariable analysis)			
Duration of isolation because of COVID-19, day	–	1.02 (1.01–1.02)	<0.001

COVID-19, coronavirus disease 2019.

a. Other variables in the multivariable model 1 are presented in Supplementary Table 3.

In model 2, compared with the control group, the no specific treatment for COVID-19 subgroup (*n* = 5256) and the specific treatment for COVID-19 subgroup (*n* = 892) had a 2.23-fold (OR = 2.23, 95% CI 2.03–2.45, *P* < 0.001) and 3.27-fold times (OR = 3.27, 95% CI 2.77–3.87, *P* < 0.001) higher prevalence of mental illness among survivors, respectively.

In multivariable model 3, a 1-day increase in the duration of isolation (in government centres and/or days spent in hospital) because of COVID-19 was associated with a 2% higher prevalence of mental illness (OR = 1.02, 95% CI 1.01–1.02; *P* < 0.001). The ORs with 95% CIs for all other variables in the multivariable model 1 are presented in Supplementary Table 3. A similar trend was observed in the sensitivity analysis, excluding 6467 individuals who died in 2020, as shown in Supplementary Table 4.

The results of the logistic regression analysis for the development of mental illness in detail are presented in Table 3. The COVID-19 survivors showed higher prevalence of affective psychotic disorders (OR = 1.44, 95% CI 1.13–1.85, *P* = 0.003), anxiety and stress-related disorder (OR = 1.72, 95% CI 1.56–1.89, *P* < 0.001) and mood disorders without psychotic symptoms (OR = 2.15, 95% CI 1.92–2.41, *P* < 0.001) than the control group.

**Table 3:** Multivariable logistic regression analysis for development of mental illness in detail

Variable	Multivariable model, odds ratio (95% CI)	*P*
Non-affective psychotic disorder		
COVID-19 survivors (versus control)	0.89 (0.52–1.55)	0.688
Affective psychotic disorders		
COVID-19 survivors (versus control)	1.44 (1.13–1.85)	0.003
Anxiety and stress-related disorder		
COVID-19 survivors (versus control)	1.72 (1.56–1.89)	<0.001
Alcohol or drug misuse		
COVID-19 survivors (versus control)	0.92 (0.54–1.56)	0.748
Mood disorders without psychotic symptoms		
COVID-19 survivors (versus control)	2.15 (1.92–2.41)	<0.001
Eating disorders		
COVID-19 survivors (versus control)	0.68 (0.17–2.75)	0.586
Personality disorders		
COVID-19 survivors (versus control)	1.55 (0.38–6.38)	0.544

COVID-19, coronavirus disease 2019.

The results of the subgroup analyses are shown in Table 4. The ORs for mental illness in the COVID-19 survivors were 1.67 (OR = 1.67, 95% CI 1.45–1.92, *P* < 0.001) in the male group and 2.06 (OR = 2.06, 95% CI 1.87–2.27, *P* < 0.001) in the female group. The ORs for mental illness in the COVID-19 survivors were 1.60 (95% CI 1.36–1.89, *P* < 0.001), 2.27 (95% CI 1.99–2.58, *P* < 0.001) and 1.86 (OR = 1.86, 95% CI 1.63–2.13, *P* < 0.001) in the 20–39, 40–59, and ≥60 years groups, respectively. Those individuals with 0–2 and ≥3 points on the CCI showed the same ORs for mental illness of 1.93.

**Table 4:** Subgroup analyses

Variable	Development of mental illness, *n* (%)	Multivariable model, odds ratio (95% CI)	*P*
Male (*n* = 140 391)			
Control	9656/137 977 (7.0)	1	
COVID-19 survivors	265/2414 (11.0)	1.67 (1.45–1.92)	<0.001
Female (*n* = 120 492)			
Control	9868/116 758 (8.5)	1	
COVID-19 survivors	473/3734 (12.7)	2.06 (1.87–2.27)	<0.001
Age: 20–39 (*n* = 109 567)			
Control	6948/106 903 (6.5)	1	
COVID-19 survivors	213/2664 (8.0)	1.60 (1.36–1.89)	<0.001
Age: 40–59 (*n* = 84 723)			
Control	5782/82 600 (7.0)	1	
COVID-19 survivors	301/2123 (14.2)	2.27 (1.99–2.58)	<0.001
Age: ≥60 (*n* = 66 593)			
Control	6794/65 232 (10.4)	1	
COVID-19 survivors	224/1361 (16.5)	1.86 (1.63–2.13)	<0.001
Charlson Comorbidity Index: 0–2 (*n* = 158 863)			
Control	11 569/156 343 (7.4)	1	
COVID-19 survivors	322/2520 (12.8)	1.93 (1.72–2.15)	<0.001
Charlson Comorbidity Index: ≥3 (*n* = 102 020)			
Control	7955/98 392 (8.1)	1	
COVID-19 survivors	416/3628 (11.5)	1.93 (1.71–2.17)	<0.001

COVID-19, coronavirus disease 2019.

## Discussion

### Main findings

This nationwide cohort study in South Korea established that, compared with the control group, COVID-19 survivors are associated with a 2.40-fold higher prevalence of mental illness, at least during the 6-month follow-up period. Among mental illnesses, a higher prevalence of mood disorders, anxiety and stress-related disorders, and affective psychotic disorders were associated with the survivors. Furthermore, this association was more evident in COVID-19 survivors who experienced specific treatment for the infection in hospitals. The study suggests that newly developed mental illness is a significant issue observed in COVID-19 survivors.

### Comparison with findings from other studies

Using the same database (NHIS COVID-19), we recently reported that COVID-19 survivors were at higher risk of psychological sequalae.^8^ However, the results of the previous study differ from those of the current study in several ways. First, the previous study was based on the database from 1 January to 4 June 2020, whereas the current study used the updated data from 1 January to 1 December 2020. Therefore, we were able to evaluate in the current study the development of mental illness across a longer period of 6 months. Second, different criteria were used for determining the primary end-point. Whereas the previous study focused on the development of depression, psychosis, alcohol misuse and drug misuse, we included more mental illnesses in this study, using the criteria used in the study by Lee et al.^15^ Therefore, anxiety and stress-related disorders, eating disorders and personality disorders were evaluated as end-points in the current study. Finally, whereas the previous study only included data on COVID-19 survivors and a control population, extracted using stratification methods, the current study included test-negative individuals as well. Thus, we examine a significantly larger sample size compared with the previous study.

In South Korea, the association of mental illness with susceptibility and outcomes of COVID-19 have been sufficiently reported,^15,17^ but information regarding the new development of mental illness among survivors was lacking. Previous studies suggest that psychological sequelae are an important issue among COVID-19 survivors.^7,9–11^ In the USA, survivors of COVID-19 have been found to experience psychiatric disorders 14–90 days after a COVID-19 diagnosis, and anxiety disorders, insomnia and dementia were mostly observed.^10^ In South Korea, the development of psychological sequelae has been recently reported in COVID-19 survivors.^9,11^ The present study was conducted during a 6-month follow-up of COVID-19 survivors to evaluate psychiatric diagnosis and is different from previous studies that evaluated mental illness during a relatively short period.^9–11^ Furthermore, unlike previous studies,^9–11^ the impact of COVID-19 severity on the development of mental illness includes information about treatment received for COVID-19.

In the competing risk analyses, a higher prevalence of mood disorders, anxiety and stress-related disorders, and affective psychotic disorders were observed in COVID-19 survivors. Depression is a major form of mood disorder, and post-traumatic stress disorder (PTSD) and anxiety disorders are major types of anxiety and stress-related disorders. A previous cohort study reported that a high prevalence of psychiatric sequelae such as PTSD, depression and anxiety were observed among 402 COVID-19 survivors at a 1-month follow-up after admission to hospital.^18^ The prevalence of mental illness among COVID-19 survivors is comparable with psychiatric sequelae for other traumatic experiences. A previous cohort study in Australia reported that 22% of people experienced a new psychiatric disorder after traumatic brain injury.^19^ Specifically, patients were more likely to develop PTSD, panic disorder, agoraphobia and social phobia significantly at 12 months after traumatic brain injury.^19^ Compared with the survivors from traumatic brain injury,^19^ we showed that a higher prevalence of mood disorder was observed in the COVID-19 survivors in our study.

Another previous cohort study in South Korea reported that 42.9% and 27.0% of Middle East Respiratory Syndrome (MERS) survivors reported PTSD and depression, respectively, at 1 year after the MERS outbreak.^20^ In a multivariable analysis, anxiety and recognition of stigma during the MERS outbreak period were significant risk factors for developing PTSD at 12 months after the MERS outbreak.^20^ Compared with the MERS survivors,^20^ the COVID-19 survivors showed a significant increase in the prevalence of psychotic disorders in our study. In 2021, Taquet et al reported that COVID-19 survivors in the USA had a 1.81-fold higher prevalence of mood, anxiety or psychotic disorder compared with those diagnosed with influenza infection at 6-months follow-up.^21^ Considering the results in our study and those of Taquet et al, the COVID-19 survivors were at a higher risk of developing mental illnesses, which includes mood, anxiety, or psychotic disorders.^21^

### Interpretation of our findings

This study establishes that COVID-19 survivors who did not require any specific treatment for infection were 2.23-fold more likely to have a mental illness than those in the control group. In South Korea, patients with COVID-19 who had mild or no symptoms were isolated with monitoring in government-managed centres until the results of the PCR test were negative. Individual stress could be an important factor leading to this phenomenon. First, the COVID-19 pandemic leads to changes in interpersonal relationships and lifestyles because of prolonged periods of solitude and loneliness. These psychosocial stressful conditions have detrimental effects on fragile individuals and affect their ability to modulate emotions.^22,23^ Second, the higher prevalence of mental illness among COVID-19 survivors might be caused by social stigma among individuals, as reported in previous studies.^24,25^ Among survivors in South Korea, social stigma during the MERS outbreak was an independent risk factor for developing PTSD after 12 months.^20^ Furthermore, a recent study in Germany reported that patients who had COVID-19 were at a higher risk of unemployment because of the social stigma attached to the infection.^26^ Such unemployment could lead to depression according to a previous epidemiological study.^27^ Therefore, this study establishes that mental health in COVID-19 survivors is a significant public health issue not only for those who were severely ill but also for patients with mild or no symptoms. However, given that we could not demonstrate the direct impact of emotional stress on mental illness development in those COVID-19 survivors with mild or no symptoms in this study, more research is needed regarding this issue.

### Limitations

This study has some limitations. First, the variables body mass index, history of alcohol consumption and smoking were not included in the adjustment as the NHIS database did not include this information. Second, the difference in sample size between the control group and the COVID-19 survivor group is of a large order of magnitude: 254 735 individuals in the control group and 6148 individuals in the COVID-19 survivor group. Third, the multivariable logistic regression is known to adjust only measured confounders, and there might be unmeasured confounders that might affect the results. Fourth, although mental illnesses should be registered in the NHIS database for individuals to receive proper financial support from the government, some data for some patients may be missing because of the lack of accessibility to medical resources among the national population in South Korea. Finally, we included information about patients who required and received specific treatment for their COVID-19 infection and used this to reflect the severity of COVID-19; an accurate measure of the severity of the COVID-19 infection was not considered in this study.

To conclude, during the approximately 6-month follow-up period, COVID-19 survivors were at a higher risk of mental illness compared with the rest of the population of South Korea. Moreover, this association was more evident in COVID-19 survivors who experienced specific treatment for the infection in hospital. To enhance the quality of life of COVID-19 survivors, proper treatment of such mental illnesses should be an important health consideration in future.

## Data Availability

Data are available upon reasonable request. Anonymised data used in the present study may be available upon reasonable request to the corresponding author.

## Supplementary material

For supplementary material accompanying this paper visit http://dx.doi.org/10.1192/bjo.2021.1001. click here to view supplementary material
