# Supplementary material for: Prevalence of mental illness among COVID-19 survivors in South Korea: nationwide cohort
Source: BJPsych Open. 2021 Oct 1;7(6):e183. doi: 10.1192/bjo.2021.1001 (PMC8503052; doi:10.1192/bjo.2021.1001)
Supplement: Supplementary file 1 [file bjosup.zip › S2056472421010012sup001.docx]

Table S2. Clinico-epidemiological characteristics of total participants (n=260,883)

| Variable | | Number (%) | Mean (SD) |
| --- | --- | --- | --- |
| Gender: male | | 119,172 (45.7) |  |
| Age | |  |  |
|  | 20-29 | 64,025 (24.5) |  |
|  | 30-39 | 45,542 (17.5) |  |
|  | 40-49 | 41,157 (15.8) |  |
|  | 50-59 | 43,566 (16.7) |  |
|  | 60-69 | 32,702 (12.5) |  |
|  | 70-79 | 19,462 (7.5) |  |
|  | ≥ 80 | 14,429 (5.5) |  |
| Residence in 2020 | |  |  |
|  | Seoul | 43,104 (16.5) |  |
|  | Gyeonggido | 46,088 (17.7) |  |
|  | Daegu | 83,502 (32.0) |  |
|  | Gyeongsangbukdo | 20,947 (8.0) |  |
|  | Other area | 67,242 (25.8) |  |
| Annual income level in 2020 | |  |  |
|  | Q1 (lowest) | 56,152 (21.5) |  |
|  | Q2 | 53,090 (20.4) |  |
|  | Q3 | 65,365 (25.1) |  |
|  | Q4 | 81,690 (31.3) |  |
|  | Unknown | 4,586 (1.8) |  |
| Underlying disability | |  |  |
|  | Mild to moderate | 10,196 (3.9) |  |
|  | Severe | 6,528 (2.5) |  |
| Charlson comorbidity index | |  | 2.7 (2.8) |
|  | Myocardial infarction | 6,624 (2.5) |  |
|  | Congestive heart failure | 19,857 (7.5) |  |
|  | Peripheral vascular disease | 38,955 (14.9) |  |
|  | Cerebrovascular disease | 25,946 (9.9) |  |
|  | Dementia | 10,863 (8.2) |  |
|  | Chronic pulmonary disease | 141,040 (54.1) |  |
|  | Rheumatic disease | 23,090 (8.9) |  |
|  | Peptic ulcer disease | 110,220 (42.2) |  |
|  | Mild liver disease | 107,130 (41.1) |  |
|  | Diabetes without chronic complication | 60,886 (23.3) |  |
|  | Diabetes with chronic complication | 19,189 (7.4) |  |
|  | Hemiplegia or paraplegia | 3,374 (1.3) |  |
|  | Renal disease | 9,542 (3.7) |  |
|  | Any malignancy | 32,220 (12.4) |  |
|  | Moderate or severe liver disease | 1,735 (0.7) |  |
|  | Metastatic solid tumour | 6,093 (2.3) |  |
|  | AIDS/HIV | 428 (0.2) |  |
| Mental illness development in 2020 | | 20,262 (7.8) |  |
|  | Non-affective psychotic disorders | 763 (0.3) |  |
|  | Affective psychotic disorders | 2,914 (1.1) |  |
|  | Anxiety and stress related disorder | 13,736 (5.3) |  |
|  | Alcohol or drug misuse | 679 (0.3) |  |
|  | Mood disorders without psychotic symptoms | 8,089 (3.1) |  |
|  | Eating disorders | 169 (0.1) |  |
|  | Personality disorders | 59 (0.0) |  |

SD, standard deviation; AIDS, acquired immunodeficiency syndrome; HIV, human immunodeficiency virus
