# Supplementary material for: Prevalence of mental illness among COVID-19 survivors in South Korea: nationwide cohort
Source: BJPsych Open. 2021 Oct 1;7(6):e183. doi: 10.1192/bjo.2021.1001 (PMC8503052; doi:10.1192/bjo.2021.1001)
Supplement: Supplementary file 1 [file bjosup.zip › S2056472421010012sup002.docx]

Table S3. Other variables in multivariable logistic regression model 1

| Other variables in model 1 | | Multivariable model | *P*-value |
| --- | --- | --- | --- |
|  |  | OR (95% CI) |  |
| Gender: male | | 0.87 (0.84, 0.90) | <0.001 |
| Age | |  |  |
|  | 20-29 | 1 |  |
|  | 30-39 | 1.14 (1.07, 1.21) | <0.001 |
|  | 40-49 | 1.35 (1.27, 1.43) | <0.001 |
|  | 50-59 | 1.75 (1.65, 1.84) | <0.001 |
|  | 60-69 | 2.19 (2.07, 2.32) | <0.001 |
|  | 70-79 | 2.85 (2.68, 3.04) | <0.001 |
|  | ≥80 | 3.82 (3.57, 4.09) | <0.001 |
| Residence in 2020 | |  |  |
|  | Seoul | 1 |  |
|  | Gyeonggido | 1.00 (0.95, 1.05) | 0.898 |
|  | Daegu | 0.55 (0.53, 0.58) | <0.001 |
|  | Gyeongsangbukdo | 0.78 (0.74, 0.83) | <0.001 |
|  | Other area | 1.17 (1.12, 1.22) | <0.001 |
| Annual income level in 2020 | |  |  |
|  | Q1 (lowest) | 1 |  |
|  | Q2 | 0.93 (0.88, 0.97) | 0.001 |
|  | Q3 | 0.89 (0.85, 0.93) | <0.001 |
|  | Q4 | 0.85 (0.81, 0.88) | <0.001 |
|  | Unknown | 0.88 (0.78, 0.99) | 0.037 |
| Underlying disability | |  |  |
|  | Mild to moderate (vs no disability) | 1.09 (1.02, 1.15) | 0.007 |
|  | Severe (vs no disability) | 1.14 (1.05, 1.23) | 0.001 |
| Charlson comorbidity index | | 1.09 (1.08, 1.09) | <0.001 |
|  | Myocardial infarction | 1.09 (1.02, 1.18) | 0.015 |
|  | Congestive heart failure | 1.05 (1.00, 1.10) | 0.044 |
|  | Peripheral vascular disease | 1.22 (1.17, 1.25) | <0.001 |
|  | Cerebrovascular disease | 1.21 (1.16, 1.27) | <0.001 |
|  | Dementia | 1.24 (1.17, 1.31) | <0.001 |
|  | Chronic pulmonary disease | 1.18 (1.14, 1.22) | <0.001 |
|  | Rheumatic disease | 1.12 (1.07, 1.17) | <0.001 |
|  | Peptic ulcer disease | 1.21 (1.17, 1.25) | <0.001 |
|  | Mild liver disease | 1.26 (1.22, 1.30) | <0.001 |
|  | Diabetes without chronic complication | 1.09 (1.05, 1.13) | <0.001 |
|  | Diabetes with chronic complication | 0.97 (0.92, 1.02) | 0.242 |
|  | Hemiplegia or paraplegia | 1.15 (1.05, 1.27) | 0.004 |
|  | Renal disease | 1.02 (0.96, 1.09) | 0.473 |
|  | Any malignancy | 1.07 (1.03, 1.12) | 0.001 |
|  | Moderate or severe liver disease | 1.13 (0.98, 1.29) | 0.091 |
|  | Metastatic solid tumour | 1.20 (1.11, 1.30) | <0.001 |
|  | AIDS/HIV | 1.31 (0.97, 1.76) | 0.078 |

OR, odds ratio; CI, confidence interval; AIDS, acquired immunodeficiency syndrome; HIV, human immunodeficiency virus
