# Supplementary material for: Prevalence of mental illness among COVID-19 survivors in South Korea: nationwide cohort
Source: BJPsych Open. 2021 Oct 1;7(6):e183. doi: 10.1192/bjo.2021.1001 (PMC8503052; doi:10.1192/bjo.2021.1001)
Supplement: Supplementary file 1 [file bjosup.zip › S2056472421010012sup003.docx]

Table S4. Logistic regression analysis for development of mental illness in 2020, excluding 6,467 individuals who died in 2020 (n=254,416)

| Variable | | Development of mental illness n, (%) | Logistic regression | *P*-value |
| --- | --- | --- | --- | --- |
|  |  |  | OR (95% CI) |  |
| Unadjusted (univariable analysis) | |  |  |  |
|  | Control | 18,620 of 248,278 (7.5%) | 1 |  |
|  | COVID-19 survivors | 734 of 6,138 (12.0%) | 1.73 (1.60, 1.87) | <0.001 |
| Covariates-adjusted model 1 (multivariable analysis) | |  |  |  |
|  | Control | 18,620 of 248,278 (7.5%) | 1 |  |
|  | COVID-19 survivors | 734 of 6,138 (12.0%) | 2.43 (2.24, 2.64) | <0.001 |
| Covariates-adjusted model 2 (multivariable analysis) | |  |  |  |
|  | Control (n=248,278) | 18,620 of 248,278 (7.5%) | 1 |  |
|  | No specific treatment for COVID-19 (n=5,255) | 524 of 5,255 (10.0%) | 2.26 (2.06, 2.48) | <0.001 |
|  | Specific treatment for COVID-19 (n=883) | 210 of 883 (23.8%) | 3.31 (2.80, 3.92) | <0.001 |
| Covariates-adjusted model 3 (multivariable analysis) | |  |  |  |
|  | Duration of isolation due to COVID-19, day |  | 1.01 (1.01, 1.02) | <0.001 |

OR, odds ratio; CI, confidence interval; COVID-19, coronavirus disease
